# Systemic angiogenic protein changes following propranolol therapy in infantile hemangioma: a multi-target perspective

**DOI:** 10.3389/fphar.2025.1706048

**Published:** 2025-12-19

**Authors:** Lijun Liang, Yan Zhang, Yan Ma, Jing Liu, Wangnan Du, Qiang Ma

**Affiliations:** 1 Department of Pediatrics, General Hospital of Ningxia Medical University, Yinchuan, China; 2 Ningxia Health Vocational and Technical College, Shizuishan, China; 3 Graduate School, Dalian Medical University, Liaoning, China; 4 The First Clinical Medical College of Ningxia Medical University, Yinchuan, China

**Keywords:** angiogenesis, biomarkers, infantile hemangioma, propranolol, signaling pathways

## Abstract

**Introduction:**

Infantile hemangioma (IH) is a common benign vascular tumor in infants, often requiring intervention due to potential functional impairment and cosmetic concerns. Propranolol, a nonselective β-adrenergic receptor blocker, is the first-line therapy for IH, yet its mechanisms remain incompletely elucidated.

**Methods:**

This prospective study investigated the systemic angiogenic protein profile changes in response to propranolol in 14 treatment-naïve IH infants compared to 14 healthy controls using antibody array analysis.

**Results:**

We identified twenty-six angiogenic proteins significantly downregulated in pretreatment IH patients compared to healthy controls. After 3 months of propranolol treatment, six proteins including HB-EGF, TGFα, ANGPTL4, Follistatin, Tie-1 and PLGF were significantly upregulated. Bioinformatic enrichment analysis revealed that these proteins are involved in key biological processes and signaling pathways, including epithelial cell proliferation, angiogenesis regulation, VEGF signaling, ERBB-EGFR axis, Ras-MAPK, and PI3K-Akt pathways.

**Discussion:**

These results suggest that propranolol treatment is associated with a rebalancing of dysregulated angiogenic proteins in IH, through modulating both pro- and anti-angiogenic factors to rebalance vascular homeostasis. Our study provides novel insights into the systems-level pharmacological actions of propranolol and proposes potential biomarkers for treatment response evaluation.

## 1 Introduction

Infantile hemangioma (IH) is one of the most common benign vascular tumors in infancy, arising from the abnormal proliferation of mesoderm-derived vascular tissue. With a high incidence rate, approximately 60% of IHs occur in the head, neck, and facial regions, often leading to significant clinical concerns due to their conspicuous locations (Bandera et al., 2021). Although IH is a benign condition, its clinical manifestations are highly variable. Beyond cosmetic disfigurement, IH can cause severe complications such as functional impairment (e.g., airway obstruction or visual disturbances), ulceration, bleeding, and, in rare cases, life-threatening conditions (Hasbani and Hamie, 2022). These complications not only threaten physical health but also impact psychological development, emphasizing the need for effective therapeutic interventions.

Current treatment strategies for IH include pharmacotherapy (e.g., beta-blockers), laser therapy, surgical excision, and interventional procedures. Among these, propranolol, a nonselective β-adrenergic receptor (AR) antagonist, has emerged as the first-line therapy due to its efficacy in inhibiting angiogenesis and promoting vasoconstriction (Lee et al., 2021). Propranolol’s therapeutic effects were discovered serendipitously, and it has since revolutionized IH management, particularly for high-risk or complicated cases (Sebaratnam et al., 2021; Macdonald et al., 2022). However, despite its widespread use, the molecular mechanisms underlying propranolol’s action remain incompletely understood.

The precise mechanism of propranolol in IH treatment is multifaceted and not solely dependent on β-AR blockade. Recent studies suggest that its R (+) enantiomer, which lacks β-blocking activity, directly targets the transcription factor SOX18, suppressing the mevalonate pathway and downstream effectors like sterol regulatory element-binding protein 2 and HMG-CoA reductase (Schrenk and Boscolo, 2022; Holm et al., 2025). Additionally, propranolol modulates metabolic pathways in hemangioma-derived endothelial cells, impacting glycolysis and cholesterol metabolism (Yang et al., 2023; Hu et al., 2021; Jiang et al., 2024). Other proposed mechanisms include the inhibition of hemangioma stem cell differentiation and disruption of angiogenic signaling (Seebauer et al., 2022; Chen et al., 2025). Nevertheless, challenges such as treatment resistance, recurrence, and variable patient responses highlight the need for further research to elucidate propranolol’s molecular targets and optimize therapeutic strategies (Chen et al., 2023). Understanding the molecular mechanisms of propranolol in IH is critical for developing targeted therapies, improving treatment efficacy, and mitigating adverse effects.

## 2 Materials and methods

### 2.1 Subjects

This prospective cohort study enrolled 14 treatment-naïve infants (aged 29 days to 1 year, including 6 males and 8 females) diagnosed with IH at General Hospital of Ningxia Medical University between July 2023 and December 2024. The diagnosis of IH was confirmed according to the 2019 Chinese Medical Association “Diagnosis and Treatment Guidelines for Hemangiomas and Vascular Malformations”. The IH infants were excluded who met the following criteria: incomplete medical records or age≤28 days; cardiac contraindications including severe myocardial impairment, cardiac failure, cardiogenic shock, bradycardia, second-to third-degree atrioventricular block, or severe congenital heart disease; hepatic or renal dysfunction; respiratory diseases such as bronchial asthma or pneumonia, or potential airway hyperresponsiveness; glucose intolerance or diabetes mellitus. Propranolol was administered orally every 12 h. The initial dose was set at half of the target dose (1 mg kg^-1^·d^-1^). If blood glucose levels and vital signs remained stable, the dose was increased to the full target dose (2 mg kg^-1^·d^-1^) after 24 h. All infants received propranolol administration within 30 min after feeding. Blood samples were collected at baseline and 3 months post-treatment. Additionally, 14 age-matched healthy infants (6 males and 8 females) were recruited from pediatric wellness visits at the same hospital. No significant differences in treatment response or clinical outcomes were observed based on sex in this cohort. The study protocol was approved by the Research Ethics Committee of Ningxia Medical University General Hospital (approval number: KYLL-2021-840), and written informed consent was obtained from all participants’ legal guardians prior to enrollment. All procedures were conducted in accordance with the Declaration of Helsinki.

### 2.2 Antibody arrays

Serum was isolated from blood samples and was analyzed for the levels of 60 angiogenic proteins using an array (RayBiotech, Norcross, GA, United States, GSH-ANG-1000). All experiments were conducted according to the manufacturer’s instructions, with the following specific quality control and standardization measures implemented to ensure reproducibility and minimize bias. Each array contained built-in positive controls (for signal normalization) and negative controls (for background subtraction), which were included in every run. Briefly, after incubation with blocking buffer for 1 hour at room temperature with gentle shaking, serum samples diluted at 2 folds with blocking buffer were added into the pools for incubation overnight at 4 °C. Then, following extensive washing, a cocktail of biotinylated antibodies was added to detect the bound angiogenic proteins for 2 h at room temperature. The arrays were then incubated with AlexaFluor 555-conjugated streptavidin for 2 h at room temperature. Fluorescent signals were captured using an InnoScan 300 scanner (Innopsys, Carbonne, France). Spot intensities were read through Mapix 7.3.1 Software and were background-subtracted locally. The spots for each angiogenic protein were designed for four replicates, any replicate with a coefficient of variation (CV) exceeding 20% was excluded from the analysis. The fluorescence intensities were calculated for average values and normalized through positive controls on the respective array to account for inter-array variability.

### 2.3 Bioinformatic enrichment analysis

Functional enrichment analysis for the Gene Ontology (GO) biological processes and Kyoto Encyclopedia of Genes and Genomes (KEGG) pathways was performed on the set of six significantly upregulated proteins using the clusterProfiler package (v4.10.0) in R. Significantly enriched terms were identified with a threshold of adjusted p-value <0.05 (Benjamini–Hochberg method).

### 2.4 Statistical analysis

Statistical comparisons between pretreatment IH patients and healthy controls were conducted using the Mann-Whitney U test method, between pretreatment and posttreatment IH patients using paired-T test with Statistical Package for Social Science Statistics version 20 (IBM Corp., Armonk, NY, United States). To account for multiple comparisons across the 60 angiogenic proteins, the False Discovery Rate (FDR) was controlled using the Benjamini–Hochberg procedure. Differences were considered statistically significant when p value <0.05 after FDR correction and a fold change threshold of <0.83 (downregulation) or >1.2 (upregulation). Data were expressed as mean ± standard deviation (SD).

## 3 Results

### 3.1 Patients

**FIGURE 1:**
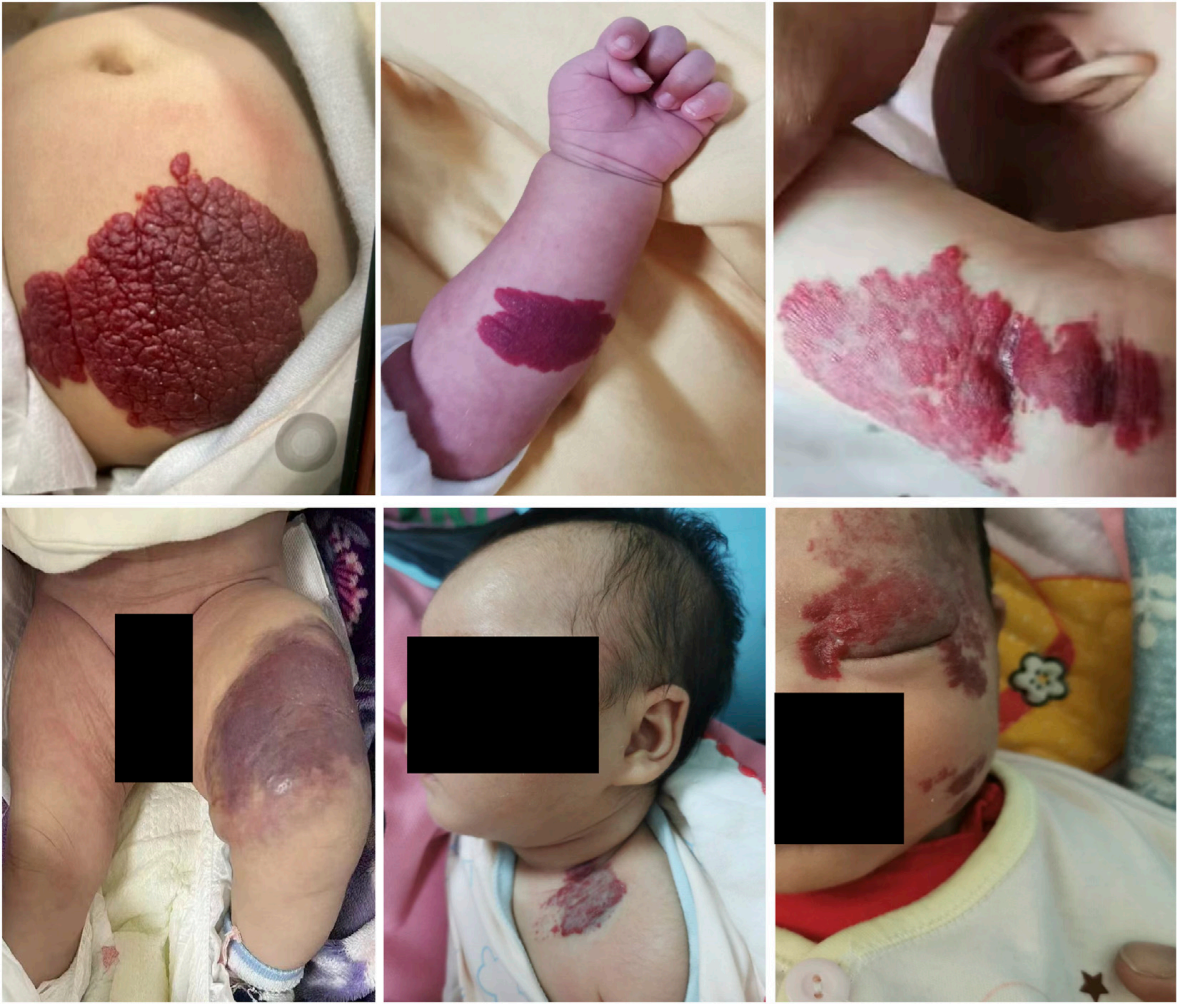
The clinical manifestations of IH patients. Hemangioma locates in different body parts of patients.

In the present study, 14 infants diagnosed as IH were referred for propranolol treatment. The follow-up period was 3 months. To ensure the safety of propranolol in the treatment of IH, a focus on monitoring indicators such as heart rate, myocardial enzymes, gastrointestinal adverse reactions (such as vomiting, diarrhea) and local reactions (such as skin ulcers) were performed. The characteristics of IH patients were shown in Table 1. The clinical manifestations of patients are shown in Figure 1.

**TABLE 1:** The characteristics of IH patients.

Demographics	H (n = 14)	Normal range
Age (months)	3.75 ± 1.64	
Male/female	6/8	
Common locations		
Head and neck (n, %)	5, 35.7%	
Trunk (n, %)	5, 35.7%	
Extremities (n, %)	4, 28.6%	
Sinus bradycardia (n, %)	0, 0%	
Digestive system		
Vomiting (n, %)	0, 0%	
Diarrhea (n, %)	0, 0%	
Decreased appetite (n, %)	2, 14.2%	
Skin rupture (n, %)	1, 7.1%	
CK (U/L)	123.1 ± 39.1	40–200
LDH (U/L)	175.9 ± 45.7	120–250
CK-MI (ng/mL)	2.5 ± 0.4	<3.38

### 3.2 A six-protein signature is upregulated following propranolol therapy

**FIGURE 2:**
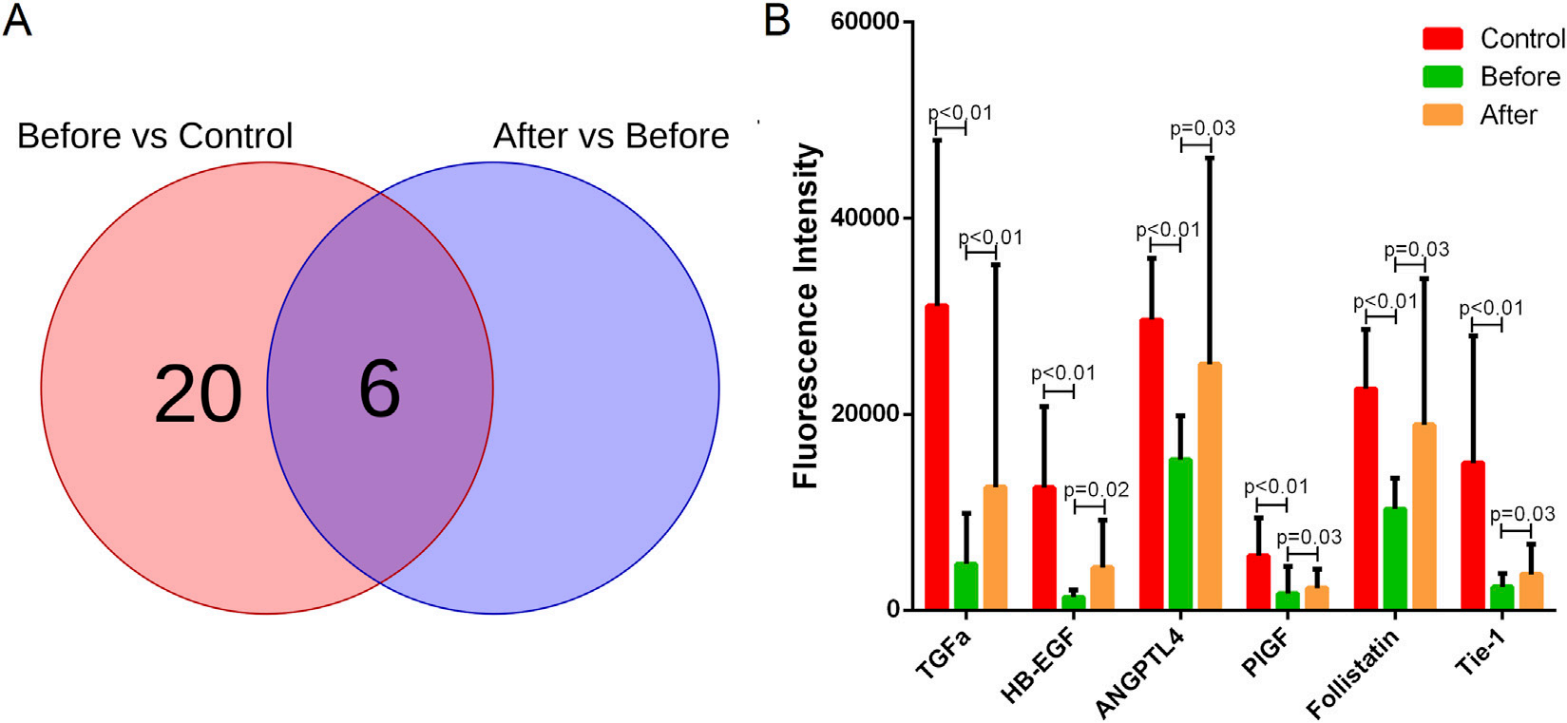
Propranolol therapy significantly upregulates six angiogenic proteins in IH. **(A)** Venn plots illustrating the differential expression profiles of angiogenic proteins between pretreatment IH patients and healthy controls (left), as well as between post-treatment and pretreatment states (right). Significantly upregulated proteins following propranolol treatment locate in the intersection. **(B)** The levels of the six markedly upregulated angiogenic proteins (Angiopoietin-like 4, Follistatin, Heparin-binding EGF-like growth factor, Placental growth factor, Transforming growth factor alpha, Tyrosine kinase with immunoglobulin-like and EGF-like domains 1) across healthy controls, pretreatment, and post-treatment groups. p values were corrected by FDR.

Comparative analysis of serum angiogenic profiles before and after propranolol treatment revealed a signature of six proteins that were significantly upregulated in response to therapy including HB-EGF, TGFα, ANGPTL4, Follistatin, Tie-1 and PLGF (Figure 2). These findings suggest that propranolol treatment is associated with a restoration of dysregulated angiogenic factor profiles, which may contribute to its therapeutic effects in IH.

### 3.3 Therapeutic efficacy evaluation

**FIGURE 3:**
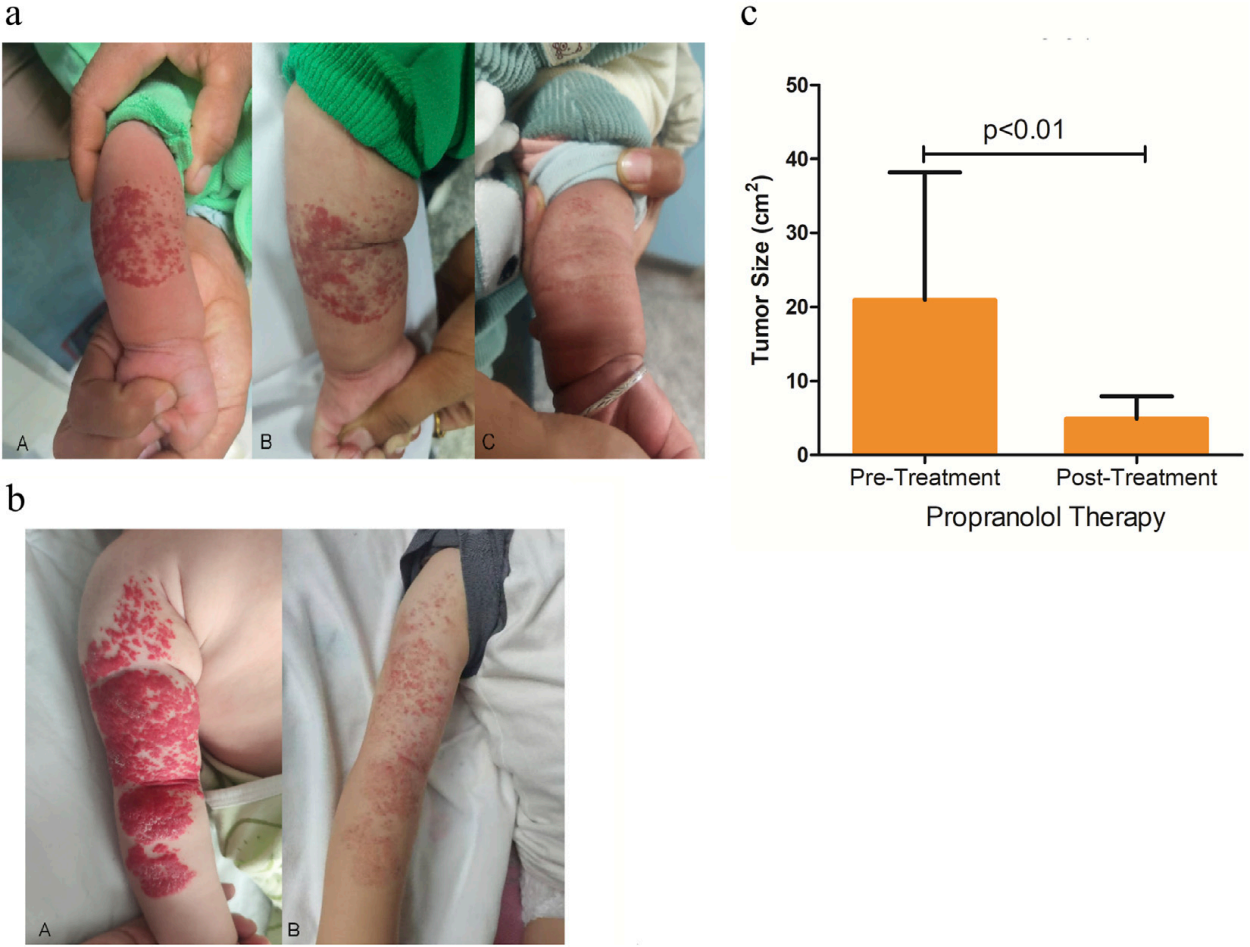
Clinical efficacy of propranolol. **(a)** Hemangioma on the left forearm. **(a)** Hemangioma presentation with 5 cm × 5 cm red plaque prior to treatment. **(b)** After 1 month of propranolol therapy, the lesion shows noticeable lightening in color. **(c)** Marked regression is observed after 3 months of treatment, with near-complete resolution of the hemangioma. **(b)** Hemangioma on the right forearm. **(a)** Hemangioma presentation with 10 cm × 5 cm red plaque prior to treatment. **(b)** After 3 months of treatment with propranolol, the skin lesions became lighter in color, thinner in thickness, and smaller in area, accompanied by pigmentation. **(c)** Quantitative analysis of tumor size before and after propranolol treatment. The bar graph shows a significant reduction in tumor area (cm^2^) following therapy. Data are presented as mean ± SD, p value <0.01 following FDR correction.

Treatment response was assessed independently by two blinded pediatric dermatologists who were not involved in patient management using both objective measurement of tumor size and the internationally recognized Achauer grading system (Achauer et al., 1997) as follows: Grade I (Poor): ≤25% reduction in hemangioma volume with no significant improvement or continued growth of skin lesions. Grade II (Moderate): 26%–50% volume reduction with arrested growth and lightening of lesion color. Grade III (Good): 51%–75% volume reduction. Grade IV (Excellent): >75% volume reduction. Grades III-IV were considered treatment success, demonstrating significant tumor regression with normalization of tissue appearance. Grade II indicated partial response with effective growth control and moderate size reduction. Grade I represented treatment failure, characterized by minimal volume reduction or therapy discontinuation due to adverse effects. All 14 patients showed significant clinical improvement, with 7 ones achieving grade III and 7 achieving grade IV, and the mean tumor size after therapy was significantly reduced, demonstrating a high rate of therapeutic efficacy (Figure 3). This marked clinical improvement, observed in all patients, may be associated with the systemic upregulation of the six-protein signature described above.

### 3.4 Dysregulated angiogenic profile in pretreated IH patients

To establish the baseline angiogenic profile in IH, we first compared serum protein levels between treatment-naïve patients and healthy controls. This analysis revealed a broad dysregulation, with twenty-six angiogenic proteins being significantly downregulated in the IH cohort, and those six proteins significantly upregulated in response to therapy were from the twenty-six angiogenic proteins (Supplementary Table S1; Figure 2A). This pre-treatment dysregulation underscores the profound abnormality of the vascular microenvironment prior to intervention. Against this backdrop, the specific upregulation of a select set of proteins following propranolol therapy becomes particularly noteworthy.

### 3.5 Bioinformatic analysis

**FIGURE 4:**
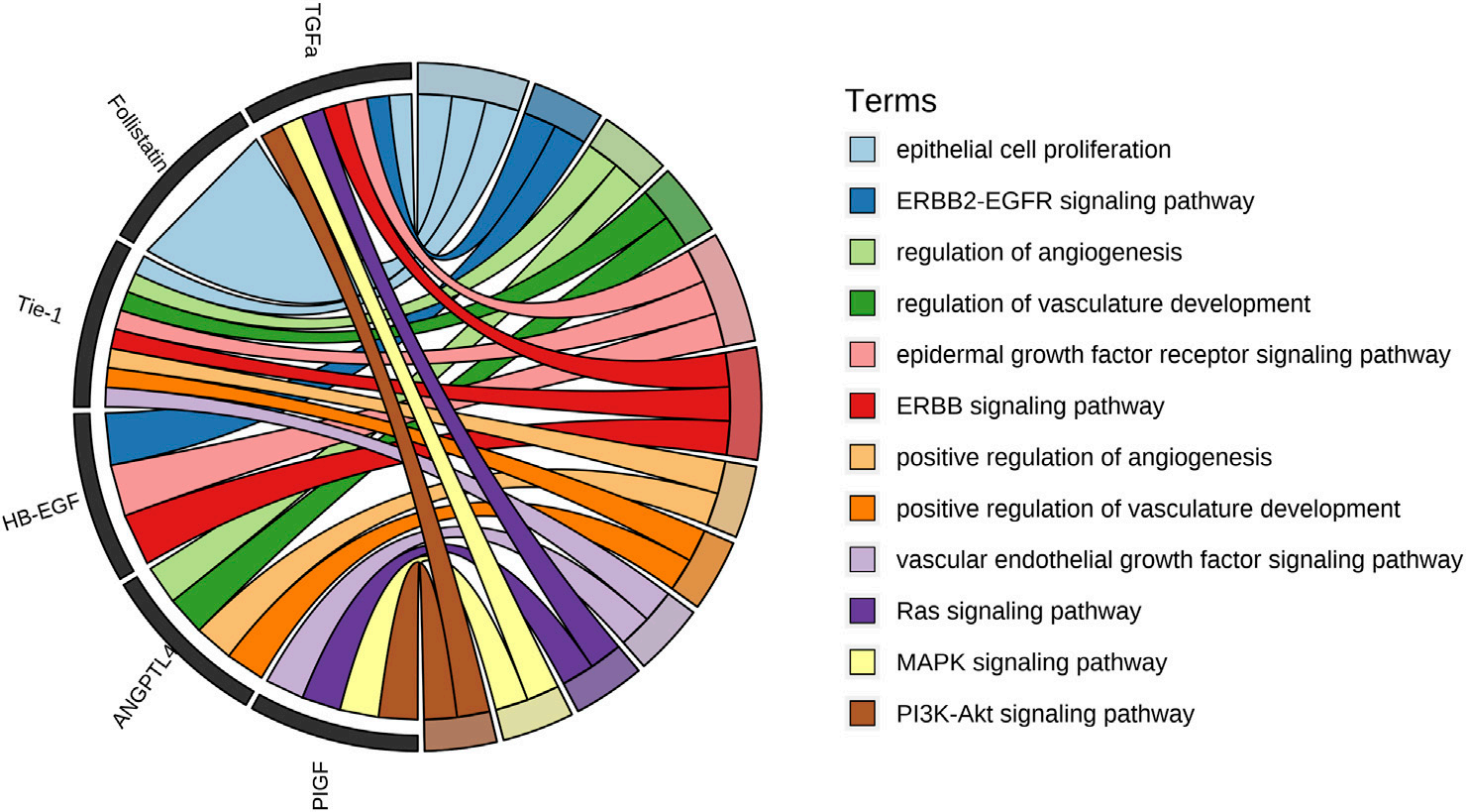
Bioinformatic enrichment analysis of the six upregulated angiogenic proteins. The chordal graph illustrates the key biological processes and signaling pathways associated with Angiopoietin-like 4, Follistatin, Heparin-binding EGF-like growth factor, Placental growth factor, Transforming growth factor alpha, Tyrosine kinase with immunoglobulin-like and EGF-like domains 1. These propranolol-upregulated proteins are significantly involved in critical functions such as epithelial cell proliferation, regulation of angiogenesis and vasculature development, and multiple signaling pathways including VEGF, ERBB2-EGFR, ERBB, Ras-MAPK, and PI3K-Akt cascades.

These six angiogenic factors were analyzed for bioinformatic enrichments to identify biological processes and signaling pathways which involve into mechanism of propranolol treatment. The essential terms include epithelial cell proliferation, ERBB2-EGFR and ERBB signaling pathways, regulation of angiogenesis and vasculature development, VEGF signaling, Ras-MAPK and PI3K-Akt signaling pathways (Supplementary Table S2), and Figure 4 shows the associated angiogenic factors involving in corresponding biological processes and signaling pathways. These findings highlight the multifaceted role of propranolol in restoring angiogenic balance, potentially through coordinated modulation of growth factor signaling and downstream pathways critical for vascular proliferation and remodeling in IH.

### 3.6 Correlation analysis of protein changes and clinical efficacy

**FIGURE 5:**
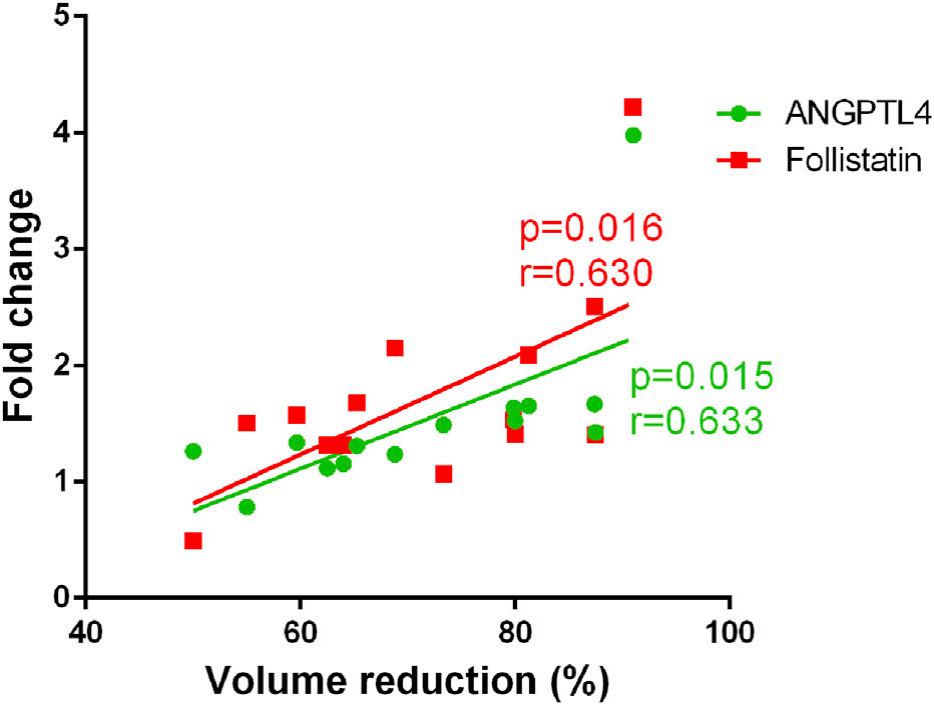
Correlation between angiogenic protein upregulation and clinical response to propranolol. Scatter plots illustrating the significant positive correlation between the fold change in serum levels of ANGPTL4 and Follistatin following propranolol treatment and the percentage reduction in hemangioma size. Statistical analysis was performed using linear regression, with correlation coefficient (r) and p value indicated on each graph.

To further elucidate whether the upregulation of the six angiogenic proteins mediates the clinical efficacy of propranolol, we performed linear regression model to analyze the relationship between reduction percentage in tumor size and fold-change of each of the six key proteins. The results revealed that the upregulation of ANGPTL4 and Follistatin were significantly and positively associated with the extent of tumor regression (Figure 5), indicating that these proteins may serve as functional biomarkers linking propranolol exposure to therapeutic response.

## 4 Discussion

Propranolol, a nonselective AR blocker, has emerged as the first-line therapy for problematic IHs, the most common benign vascular tumors in children. Despite its clinical efficacy, the precise mechanisms underlying its therapeutic effects remain incompletely understood. Current evidence suggests that propranolol acts through both β-AR-dependent and independent pathways, including modulation of angiogenic factors, metabolic reprogramming, and targeting of transcription factors like SOX18 and the mevalonate pathway (Schrenk and Boscolo, 2022; Thaler et al., 2023; Holm et al., 2025; Yang et al., 2023). However, the broader impact of propranolol on angiogenic protein networks in IH is still being elucidated.

Our study revealed that twenty-six angiogenic proteins were significantly downregulated in pretreatment IH patients compared to healthy controls. Following propranolol therapy, six proteins including HB-EGF, TGFα, ANGPTL4, Follistatin, Tie-1 and PLGF exhibited significant upregulation. This observed protein expression pattern suggests that propranolol’s therapeutic effects may be mediated through coordinated modulation of multiple angiogenic factors, not only by direct β-adrenergic receptor blockade but also by rebalancing the aberrant angiogenesis network. The upregulation of these specific proteins following therapy suggests their potential involvement in the treatment response and supports their further investigation as biomarkers.

Excessive angiogenesis is a common feature of all IHs, closely related to the growth and regression of IH (Kong et al., 2023; Xiang et al., 2024). Angiogenesis is stimulated by proangiogenic factors, characterized by more pronounced behavioral plasticity of endothelial cells (Eelen et al., 2015). Among these six angiogenic proteins, studies show that HB-EGF, TGFα, PLGF and Tie-1 exert pro-angiogenic functions, while Follistatin is known as anti-angiogenic factors (Rao et al., 2020; Le et al., 2019; Zare et al., 2023; Raja Xavier et al., 2024; Janik et al., 2019; Meltzer et al., 2022). The role of ANGPTL4 in vascular biology is complex, with both pro- and anti-angiogenic functions (Ramos et al., 2025). These six proteins were detected with lower circulating levels in IH patients compared to healthy populations in the present study, similarly Ben Li. et al. also found that HB-EGF, PLGF and Follistatin had lower levels in peripheral artery disease which is also induced by angiogenesis (Li et al., 2023). Also, TGFα was found a downregulated expression in circulating mesenchymal stromal cells in IH patients, and TGFα was upregulated after propranolol therapy (Abbà et al., 2024). In contrast, Sasaki. et al. found ANGPTL4 were increased in hemangioma stem cells and decreased following propranolol therapy (Sasaki et al., 2019). It may stem from fundamental differences in experimental context. Our study measured systemic protein levels in IH patients during therapeutic regression, whereas Sasaki et al. analyzed cellular gene expression in proliferating hemangioma stem cells. Furthermore, ANGPTL4 plays dual roles in both pro- and anti-angiogenesis, and is a secreted tumor suppressor that inhibits angiogenesis (Okochi-Takada et al., 2014), and we speculate that ANGPTL4 may also play a role in inhibiting angiogenesis in propranolol therapy. In addition, Tie-1 functions as not only a pro-angiogenic factor, but also a tyrosine kinase receptor critical for vascular stability (Wang and Zhang, 2025). Its upregulation may reflect propranolol’s stabilization of immature IH vasculature in present study.

Moreover, these proteins upregulated following propranolol therapy converge on key pathways dysregulated in IH including epithelial cell proliferation, regulation of angiogenesis and vasculature development, ERBB2-EGFR and ERBB signaling pathways, VEGF signaling, Ras-MAPK and PI3K-Akt signaling pathways. ERBB signaling has been shown to induce activation of RAS/MAPK and PI3K/Akt pathways (Zong et al., 2017). The activation of Ras-MAPK and PI3K-Akt signaling pathways triggered by VEGF signaling leads to vascular endothelial cell proliferation, cytoskeletal rearrangement and vascular penetration to mediate vasoconstriction in hemangioma (Mao et al., 2021). Taken together, propranolol may inhibit epithelial cell proliferation, angiogenesis and vasculature development in IH treatment by the modulation of these critical pathways, but this warrants further investigation, since the observed pathway enrichment may reflect complex network cross-talk or feedback mechanisms.

## 5 Conclusion

In summary, our findings suggest that propranolol’s therapeutic effects in infantile hemangioma are associated with a multifaceted modulation of angiogenic protein networks, extending beyond simple β-adrenergic receptor blockade. By upregulating a set of key angiogenic proteins including HB-EGF, TGFα, ANGPTL4, Follistatin, bFGF, Tie-1 and PLGF, propranolol restores balance to the dysregulated angiogenesis network characteristic of IH. These proteins, with their diverse and sometimes dual roles in vascular biology, are implicated in critical signaling pathways such as VEGF, ERBB, Ras-MAPK, and PI3K-Akt, ultimately contributing to the inhibition of aberrant endothelial proliferation and promotion of vascular stabilization. This systems-level mechanism not only deepens our understanding of propranolol’s action but also highlights potential biomarkers for treatment monitoring. Further functional studies are warranted to validate the causal roles of these proteins and pathways in propranolol-mediated regression of IH. However, this is an exploratory study and is limited by its single-center design and relatively small sample size, which may affect the generalizability of our findings, such as meaningful subgroup analyses based on demographic factors such as age and gender. Furthermore, the identified protein signatures, while promising, require functional validation to elucidate their precise mechanistic roles. To address these limitations, we pursue a multi-center study with an expanded cohort which not only validates these results but also enables robust subgroup analyses to investigate the impact of age, gender, and hemangioma subtypes on the proteomic response to propranolol, and employ functional assays (including cell and animal experiments) in further research.

## Data Availability

The raw data supporting the conclusions of this article will be made available by the authors, without undue reservation.

## Supplementary material

SUPPLEMENTARY TABLE 1 The raw data of sixty angiogenic proteins from antibody array.

SUPPLEMENTARY TABLE 2 The list of enrichment terms.
